# Early childhood circumstances and educational wellbeing inequality among tribal and non-tribal children in India: evidence from a panel study

**DOI:** 10.1038/s41598-022-13889-5

**Published:** 2022-06-14

**Authors:** Rashmi Rashmi, Ronak Paul

**Affiliations:** 1 Department of Population and Development, International Institute for Population Sciences, Mumbai, 400088 India; 2 Department of Public Health and Mortality Studies, International Institute for Population Sciences, Mumbai, 400088 India

**Keywords:** Psychology, Risk factors

## Abstract

Despite efforts towards bridging the education gap between tribal (Scheduled Tribe) and non-tribal (Non-Scheduled Tribe) children, contrasting poor-quality education questioned the tribal children’s educational wellbeing in India. Early childhood circumstances render a remarkable impact on the educational wellbeing of children in later years. This study examined the influence of early childhood circumstances (child, household and community characteristics) during 2005 on the educational wellbeing inequality (among India’s tribal and non-tribal children) during 2012 using the India Human Development Survey panel dataset of 8611 children. The Educational wellbeing score was obtained from reading, mathematical and writing test scores using Principal Component Factor Analysis. We performed the Blinder-Oaxaca decomposition of the educational wellbeing inequality among India’s tribal and non-tribal children. The ST children’s average educational wellbeing score (−0.41) was much lower than the Non-ST children (0.04). Findings from the Blinder-Oaxaca decomposition show that the household economic condition in children’s early ages contributed to 24% of educational wellbeing inequality among tribal and non-tribal children. Further, the education status of males and female adults and the sanitation condition of families considerably impacted educational wellbeing. The present study concludes that caste antagonism has not reduced with time. The missing focus on the minority groups resulted in a deteriorated educational wellbeing.

## Introduction

Indian society is a glorious heritage of varied cultures, languages and social identities. Such rich diversity has provided many blessings but, at the same time, brought significant challenges from the past. The insurance of providing equal educational opportunities is one such difficulty which government of India had to face while uplifting the lives of every individual^1^. Notably, in the case of the Scheduled Tribes population, imparting education was a serious concern due to cultural and geographical isolation^2^. Historically, Scheduled Tribes were termed as ‘depressed classes’, and ‘backward classes’ and mainly were isolated from the rest of the Indian society due to embedded caste and social hierarchies^3^. Such terms were further replaced, and the government renamed these communities as tribals who were protected and aided with particular interventions, starting with the enforcement of Article 342 of the Constitution of India. Traditionally, these tribal groups reside in remote areas, close to nature^4^. Unfortunately, the fast-moving modernization from cities to outskirts has resulted in a massive encroachment, resulting in displacement and leaving them exploited and poor^5^.

Education was the secondary issue for the Scheduled Tribes population as they usually struggled to fulfil their basic livelihoods needs due to continuous economic exploitation by non-tribals^5^. However, understanding the importance of education in uplifting lives and capital formation, the government of India started different initiatives like ashram schools or residential schools exclusively for ST children to educate and integrate them into mainstream society^6^. In the 1970s, the concept of ashram schools was initiated to overcome the structural barriers of tribal children in acquiring elementary to higher schooling education. Unfortunately, the poor quality of education and exclusion of the history and socio-cultural lives of Scheduled tribes’ communities in the curriculum demoralize the families to send their children^6^. Moreover, their dropout rates were very high among those who went to ashram schools. So, despite the continuous government efforts, the gap between tribal and non-tribal populations did not narrow down in these years due to discrimination, brutal suppression, and economic exploitation^7^. As a result, today, Scheduled Tribes constitute 9% of the total population, with literacy rates of merely 59% in 2011, which is much below India’s total literacy rate of 74%^8^.

One study from the tribal district of Dang in the Gujarat state of India shows that common toilet facilities for girls and boys were the standard issue for preventing the tribal girls from re-enrolling after primary schooling (beyond the fifth standard)^9^. Moreover, higher dropout was observed among schools if the instruction medium differed from the vernacular dialect of tribal children^9^. Another study from the Eastern Indian states of Jharkhand and West Bengal found that the dropout rates were high in tribal children due to economic hardship, especially during the cultivation period when the children helped their families with sowing and weeding plantation and harvesting activities^10^. In 2003, one study exploring multiple issues of primary education of tribal children of West Bengal concluded that poor infrastructure, shortage of schools and teachers, financial constraints in families, the rising market of private tuition had restricted the growth of tribal children^11^.

Though recent years have seen little success in bringing ST children to schools, the availability of such poor-quality education and problems with accessibility has constantly questioned their educational wellbeing. A cross-sectional study from India Human Development Survey 2005 shows that the Brahmin and high caste children enjoyed a higher competence in reading, writing and mathematical skills than their Dalit and tribal counterparts^12^. Besides the various factors mentioned in past research evidence, early life circumstances can also play a detrimental role in an individual’s development, especially for the ST population, deprived and marginalized in Indian society. Ample evidence shows that experience of conflicts, parent’s socioeconomic status, parental education, household condition and health condition in childhood have a persistent effect on the individual’s education^13–17^. Studies have shown that growing up with a low socioeconomic background is highly associated with lower achievements and job discontinuation in adulthood^18^. Being dependent on forests and natural resources can exert constant wealth shocks on the tribal population leading to absenteeism, dropout, stagnation that can further affect their educational wellbeing.

Using a panel dataset, this study examines to what extent early life circumstances (child, parents and household characteristics at 1–4 years of age) lead to a differential in educational wellbeing among tribal and non-tribal children (aged 8–11 years) in India. The rationale of such analysis is as follows. First, although the enrolments have increased in the last decade, difficulties in acquiring education among India’s tribal population persist. While education has helped eradicate the caste and hierarchy system in India, there reside few tribal populations which are isolated culturally and geographically, limiting the government to achieve the goal of universal elementary education in India. Moreover, the tribal children moving to schools for better opportunities are often restricted due to discrimination and exploitation. Second, while the government initiatives had increased the reach of children to schools, a prominent factor like quality education is often questioned in the form of deteriorated educational wellbeing rates among tribal children. Past evidence provided a clear picture of the poor schooling quality of tribal children. It forced us to think about the factors responsible for the educational wellbeing inequality among tribal and non-tribal children. Third, along with the inequality due to caste, one of the significant determinants, i.e., early life experiences of tribal children, must be considered due to their vulnerable and marginalized place of origin. Most brain development occurs during early childhood, and experiencing toxic stress during this period hampers educational wellbeing in later years^16,19,20^. Therefore, using a panel dataset, the present study explores the long-term educational implications of the early childhood circumstances among India’s tribal and non-tribal children. Our primary objective is to determine the long-term contribution of early childhood covariates to the inequality in educational wellbeing attainment among tribal and non-tribal children in India.

## Methods

### Data

**Figure 1:**
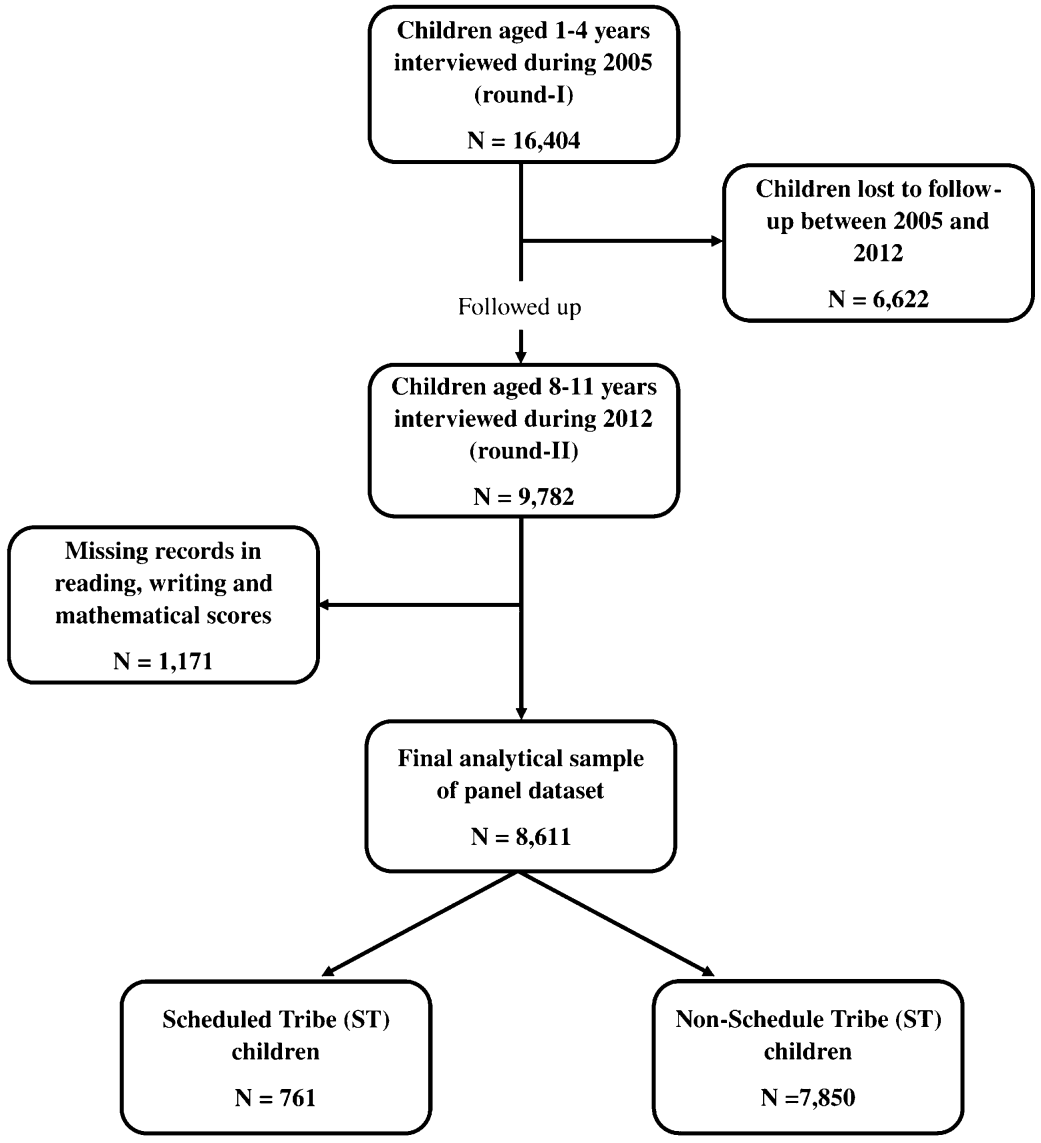
Flowchart demonstrating analytic sample based on IHDS round I (2005) and round II (2012).

The India Human Development Survey (IHDS) rounds -I and -II were used in this study. The 2005 IHDS round-I was a nationally representative multi-topic survey of 215,754 people from 41,554 households^21^. Round-II, conducted in 2012, was a multi-topic panel survey of 204,569 people from 42,152 households in India^22^. The University of Maryland, USA and the National Council of Applied Economics Research (NCAER), India, conducted the two IHDS rounds in India’s states and union territories (except Andaman and Nicobar Islands and Lakshadweep). Round-II of the IHDS re-interviewed 83% of the original families from round-I living in the same village. IHDS used a stratified random sampling design to choose samples. More information on the sampling design, survey timeframe, and data collection methods used in rounds I and II can be found elsewhere^23,24^. The analytical sample is the panel of 8611 children aged 1–4 years in round-I who became 8–11 years old during round-II, after excluding the missing observations (see Fig. 1). Of the 8,611 children, 7850 (91%) and 761 (9%) belonged to the Non-ST and ST caste groups.

### Constructing the educational wellbeing index

The continuous indicator of educational wellbeing during round-II is the outcome variable of this study. The mathematical, reading and writing test scores of children aged 8–11 years were used to prepare the educational wellbeing score.

The reading skill of students has five categories:

1. cannot read at all (score 0),
2. can read alphabets but not words (score 1),
3. can read words but cannot read entire sentences (score 2),
4. can read a short paragraph but cannot read a whole page (score 3),
5. can read a complete story (score 4).

Equivalently, the mathematical skill of students has four categories –

1. cannot read numbers (score 0),
2. can recognize numbers but cannot do any arithmetic operations (score 1),
3. can subtract a two-digit number from another number (score 2),
4. can divide a three-digit number by a one-digit number (score 3).

The writing skill of students is categorized:

1. cannot write at all (score 0),
2. can write a sentence with two or fewer mistakes (score 1).
3. can write with no mistakes (score 2).

The educational wellbeing variable was constructed using Principal Component Factor Analysis (PCFA) on the reading, mathematical, and writing skill variables and the detailed procedure is described elsewhere^25^. Notably, the PCFA for educational wellbeing indicators resulted in a one-factor solution. From supplementary Table S1, we observed that the first factor had an eigenvalue of more than one and explained 73.4% of the total variability of all three educational wellbeing indicators. All three indicators had factor loading values of more than 0.80. Further, all indicators had Kaiser-Meier-Olkin (KMO) values greater than 0.70, thereby justifying our use of PCFA (KMO values greater than 0.50 are necessary for conducting PCFA). Finally, we generated the standardized educational wellbeing score based on the first factor.

### Group variable

**Figure 2:**
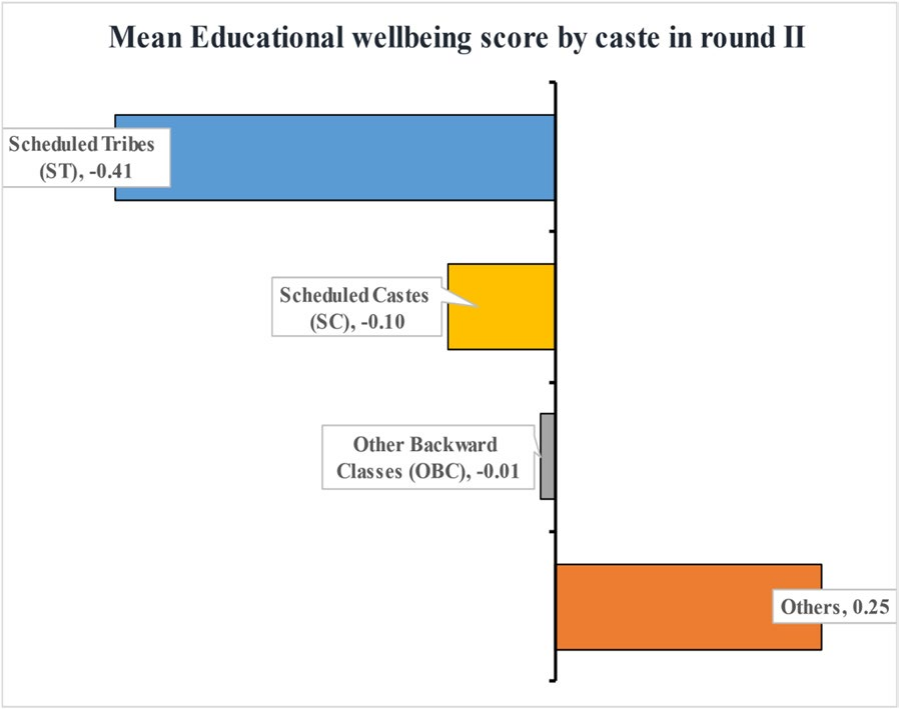
Mean educational wellbeing score of Indian children aged 8–11 years during IHDS round-II by caste groups in India.

**Figure 3:**
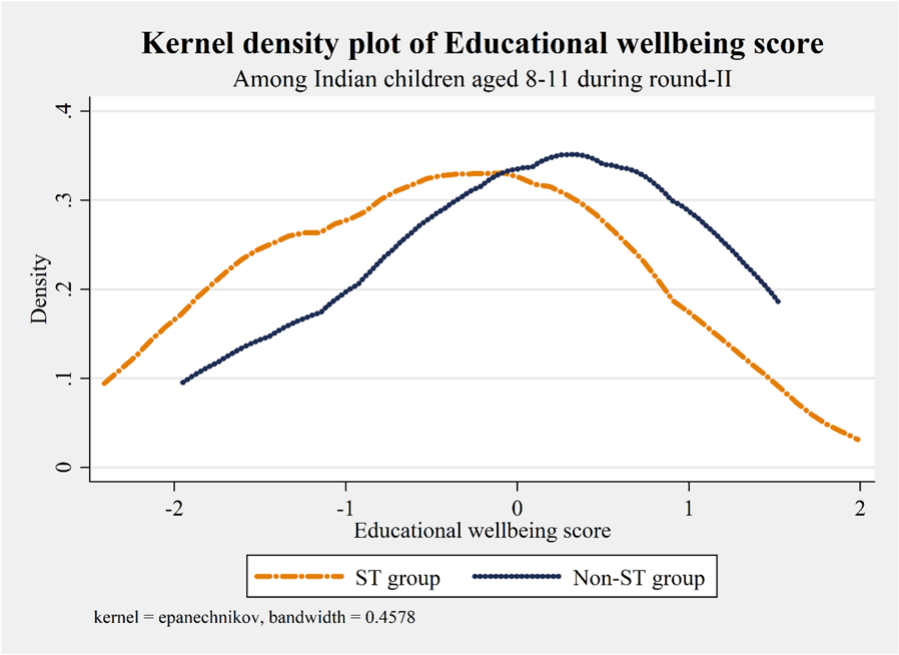
Density distribution of educational wellbeing score of ST and Non-ST Indian children aged 8–11 years during IHDS round-II.

The binary caste group variable, whether an individual belongs to the Scheduled Tribes (ST) or Non-ST, is the group variable. The caste system is a form of social hierarchy native to India. Notably, the Indian constitution recognizes three distinct social groups—Scheduled Tribes, Scheduled Castes and Other Backward Classes. People in the ST (predominantly tribal population) and SC categories are the most socially backward. They traditionally belonged to the lowest rung of India’s now-defunct caste system. People of the OBC category, as the name implies, are also members of a socially and economically backward community. However, their circumstances are better than those of the SC/ST population. The “Others” category consists of all people who do not belong to the three caste groups. During round-II, IHDS classified the caste of the household head into five categories—Brahmin, Other Backward Classes (OBC), Scheduled Castes (SC), Schedule Tribes (ST), Others. In this study, we re-coded the original variable into ST and Non-ST groups because the ST children’s educational wellbeing is markedly lower than the Non-ST children during round-II (see Figs. 2 and 3).

### Explanatory variables

Taking a cue from extant research, we included the child-, household- and community-related independent variables which explained educational wellbeing in Indian children^16,26–28^. All the variables were obtained for children aged 1–4 years in round-I unless mentioned otherwise. The child-related explanatory characteristics are:

1. Age of children in years (One, Two, Three, Four).
2. Gender of the children (Female, Male).
3. Stunting status of children (Stunted, Not stunted). Stunting indicates chronic undernutrition in children and is denoted by low height-for-age z-scores (HAZ)^29^. Although IHDS does not provide readymade HAZ scores, we obtained the HAZ scores of children aged 1–4 years in 2005 from their anthropometric data and the WHO Anthro software^30^. Children with HAZ scores of less than −2 standard deviations (SD) and more than − 6SD were coded as “Stunted”, and those having HAZ scores of more than − 2SD and less than + 6SD were coded as “Not stunted”.
4. Type of school attended in round-II (Public school, Private school).
5. Takes private tuition in round-II (No, Yes).

The household-related explanatory characteristics are:

(6) Place of cooking in the household (Not in living area, In living area).
(7) Type of cooking fuel (Solid fuel, Clean fuel).
(8) Household sanitation condition (Poor, Average, Good). Based on extant studies, the sanitation condition of the household was prepared from the information on the type of drinking water, type of toilet facility and the number of members per room during 2005^16,31^. Households having “improved” drinking water and toilet facilities were scored as “1”, and households in the “unimproved” counterpart were scored as “0”. Equivalently, households with less than three members per room were scored as “1” and “0” otherwise. We added scores of the three variables to obtain household sanitation scores ranging from 0 to 3. Households with a score of 3, 2, or less than 2 were classified as “good,” “average,” or “poor” sanitation households, respectively.
(9) Water purification in the household (No, Yes).
(10) Household wealth quintile (Poorest, Poor, Medium, Rich, Richest). The household wealth quintile variable was constructed using standard procedures documented elsewhere^32,33^. We generated wealth scores by applying principal component factor analysis (PCFA) on the household asset ownership, livestock ownership, and type of building material information during 2005. The households were then classified into five wealth quintiles from “poorest” to “richest” based on the wealth scores.
(11) Household poverty status (Below poverty line, Not below poverty line).
(12) Highest educational level of male adults in household (No formal schooling, Upto 5 years of schooling, 6–10 years of schooling, More than 10 years of schooling). IHDS 2005 provided information for years of schooling for each adult (aged 21 years and above), aggregated to obtain the highest year of schooling among all male adults in a household. Based on general milestones in the Indian education system, we further recategorized the information on years into four classifications.
(13) Highest educational level of female adults in household (No formal schooling, Upto 5 years of schooling, 6–10 years of schooling, More than 10 years of schooling). The construction of this variable is similar to the highest educational level of male adults in the household.
(14) Gender of household head (Male, Female).
(15) Religion of household head (Hindu, Muslim, Others).
(16) Types of mass media viewed by children (None, One, Two or more).
(17) Women’s autonomy in child healthcare in the household (No, Yes).
(18) Attack/threat on household (Yes, No).

The community-related explanatory characteristics are:

(19) Solving community problems (Each family individually, All families together).
(20) Domestic violence in the community (Yes, No). IHDS 2005, collected information from a single woman (aged 15–49 years) from each household on whether husbands in the community assaulted their wives if—“her natal family does not provide money, jewelry and other items”, “she does not cook food properly”, “she goes out without telling him”, “she neglects the house or the children” and “is suspected of having a relationship with other men”. If a woman responded positively to any of the five questions, we classified domestic violence status in the community as “Yes” or a “No” otherwise.
(21) Type of community (Urban, Rural).
(22) Country regions (Northern, North-eastern, Central, Eastern, Western, Southern). We divided India’s erstwhile 33 states and union territories into six regions based on administrative classification and geographical location^34^.

### Statistical methods

At the start, we showed the absolute and percentage distribution of children by the background characteristics in round-I. The caste difference in average educational wellbeing score was assessed using the two-sample t-test. The caste difference in educational wellbeing across the explanatory variables was assessed using the chi-square test for independence. Next, we estimated multivariable linear regression models to examine the association between child-, household- and community-related variables in 2005 with the educational wellbeing of children in 2012. The coefficient in the multivariable models gives the adjusted change in the educational wellbeing score in round-II of children belonging to a particular category of an explanatory variable in round-I after adjusting for the effect of all the other explanatory variables^35^. The above analyses were performed separately for ST and Non-ST children.

Further, we used the Blinder-Oaxaca twofold decomposition technique to identify the contribution of explanatory covariates in 2005 behind the caste differential in the educational wellbeing of children in 2012^36^. We show the overall and detailed decomposition of the caste differential in educational wellbeing. In the overall decomposition, the caste gap in educational wellbeing is decomposed into an explained (E) component and an unexplained (C) component^36^. The detailed decomposition shows the relative contribution of each child-, household- and community-related early childhood characteristics to caste inequality in educational wellbeing during 2012.

Note that the Non-ST group is heterogeneous (as it comprises SC, OBC and Others groups), and the results of the decomposition estimates might vary if we compare the ST children’s educational wellbeing with that of SC, OBC and Others children individually. Therefore, we decomposed the educational wellbeing gap between ST and SC children, ST and OBC children, and ST and Others children, respectively. We performed this sensitivity analysis to check the sensitivity of the decomposition estimates (shown in Table 4) to the categorization of comparison groups. In our tests, none of the multivariable models violated the assumption of multicollinearity^37^. STATA software version 14 was utilized for all statistical estimations^38^.

## Results

### Descriptive statistics

Table 1 revealed the distribution of the panel of children aged 1–4 years by socio-demographic, health, household and community-related characteristics. Approximately one in every ten children were from the Scheduled Tribes group, and 47% were females. ST children’s mean educational wellbeing score (−0.41) was significantly lower than that of Non-ST children (0.04). Nearly 53% and 46% of ST and Non-ST kids were stunted. The level of private schooling was higher in non-tribal children (38%) than in their tribal counterparts (15%). Moreover, only 10% of tribal kids took tuition compared to 24% of non-tribal kids. Further, most ST children come from families who cooked using solid fuel (95%), and nearly 71% of ST children belonged to households having poor sanitation conditions and no means of water purification. Three-fourths of the ST children were from the poor-poorest wealth quintile households, and six in ten ST kids were from households below the poverty line (BPL). In the scheduled tribe population, most of the household males were uneducated and unfortunately, the figure doubles in the case of females. The male-headed household was prominent in the ST group (94%), and the presence of violence was almost 80% in the community of the ST group. Nearly 90% of the tribal children resided in a rural community, and 32% lived in the central regions of India.

**Table 1:** Socio-demographic, health-related, household and community characteristics profile of the panel of children aged 1–4 years during IHDS round-I.

Characteristics	Panel of children aged 1-4 in round-I						
	All children		ST group		Non-ST group		Caste difference
	Mean/N	SD/%	Mean/N	SD/%	Mean/N	SD/%	p-value
**Educational wellbeing score**	0.00	1.00	− 0.41	0.98	0.04	0.01	< 0.001
**Caste of household head**							
ST group	761	8.8	–	–	–	–	
Non-ST group	7850	91.2	–	–	–	–	
**Age of children (in years)**							
One	1496	17.4	127	16.7	1369	17.4	0.674
Two	2474	28.7	232	30.5	2242	28.6	
Three	2671	31.0	236	31.0	2435	31.0	
Four	1970	22.9	166	21.8	1804	23.0	
**Gender of the children**							
Female	4082	47.4	357	46.9	3725	47.5	0.776
Male	4529	52.6	404	53.1	4125	52.5	
**Stunting status of children**							
Stunted	3975	46.2	404	53.1	3571	45.5	< 0.001
Not stunted	4636	53.8	357	46.9	4279	54.5	
**Type of school attended in round-II**							
Public School	5483	63.7	646	84.9	4837	61.6	< 0.001
Private School	3128	36.3	115	15.1	3013	38.4	
**Takes private tuition in round-II**							
No	6616	76.8	684	89.9	5932	75.6	< 0.001
Yes	1995	23.2	77	10.1	1918	24.4	
**Place of cooking in household**							
Not in living area	6732	78.2	473	62.2	6259	79.7	< 0.001
In living area	1879	21.8	288	37.8	1591	20.3	
**Type of cooking fuel**							
Solid fuel	7432	86.3	725	95.3	6707	85.4	< 0.001
Clean fuel	1179	13.7	36	4.7	1143	14.6	
**Household sanitation condition**							
Poor	4550	52.8	543	71.4	4007	51.0	< 0.001
Average	2890	33.6	190	25.0	2700	34.4	
Good	1171	13.6	28	3.7	1143	14.6	
**Water purification in household**							
No	6133	71.2	536	70.4	5597	71.3	0.614
Yes	2478	28.8	225	29.6	2253	28.7	
**Household wealth quintile**							
Poorest	1884	21.9	408	53.6	1476	18.8	< 0.001
Poor	1694	19.7	179	23.5	1515	19.3	
Medium	1719	20.0	89	11.7	1630	20.8	
Rich	1759	20.4	46	6.0	1713	21.8	
Richest	1555	18.1	39	5.1	1516	19.3	
**Household poverty status**							
Below poverty line	2782	32.3	456	59.9	2326	29.6	< 0.001
Not below poverty line	5829	67.7	305	40.1	5524	70.4	
**Highest educational level of male adults in household**							
No formal schooling	1989	23.1	299	39.3	1690	21.5	< 0.001
Upto 5 years of schooling	1343	15.6	155	20.4	1188	15.1	
6-10 years of schooling	2307	26.8	164	21.6	2143	27.3	
More than 10 years of schooling	2972	34.5	143	18.8	2829	36.0	
**Highest educational level of female adults in household**							
No formal schooling	3893	45.2	478	62.8	3415	43.5	< 0.001
Upto 5 years of schooling	1273	14.8	112	14.7	1161	14.8	
6-10 years of schooling	1727	20.1	104	13.7	1623	20.7	
More than 10 years of schooling	1718	20.0	67	8.8	1651	21.0	
**Gender of household head**							
Male	8074	93.8	715	94.0	7359	93.7	0.819
Female	537	6.2	46	6.0	491	6.3	
**Religion of household head**							
Hindu	6791	78.9	604	79.4	6187	78.8	< 0.001
Muslim	1293	15.0	6	0.8	1287	16.4	
Others	527	6.1	151	19.8	376	4.8	
**Types of mass media viewed by children**							
None	3401	39.5	428	56.2	2973	37.9	< 0.001
One	3148	36.6	218	28.6	2930	37.3	
Two or more	2062	23.9	115	15.1	1947	24.8	
**Women’s autonomy in child healthcare in household**							
No	6073	70.5	580	76.2	5493	70.0	< 0.001
Yes	2538	29.5	181	23.8	2357	30.0	
**Attack/threat on household**							
Yes	230	2.7	19	2.5	211	2.7	0.755
No	8381	97.3	742	97.5	7639	97.3	
**Solving community problem**							
Each family individually	3572	41.5	330	43.4	3242	41.3	0.270
All families together	5039	58.5	431	56.6	4608	58.7	
**Domestic violence in community**							
Yes	7221	83.9	606	79.6	6615	84.3	0.001
No	1390	16.1	155	20.4	1235	15.7	
**Type of community**							
Urban	2281	26.5	77	10.1	2204	28.1	< 0.001
Rural	6330	73.5	684	89.9	5646	71.9	
**Country regions**							
Northern	3323	38.6	101	13.3	3222	41.0	< 0.001
North Eastern	198	2.3	64	8.4	134	1.7	
Central	1179	13.7	249	32.7	930	11.8	
Eastern	1522	17.7	144	18.9	1378	17.6	
Western	1097	12.7	131	17.2	966	12.3	
Southern	1292	15.0	72	9.5	1220	15.5	
**Overall**	8611	100.0	761	100.0	7850	100.0	

(a) *N* sample size, *SD* standard deviation, %: column percentage; (b) difference in educational wellbeing score by caste group was tested using T-test while the caste difference of explanatory variables was tested using the chi-square test for independence.

We checked the percentage distribution of children by relevant demographic and socioeconomic characteristics in the cross-sectional and panel survey during the baseline period for possible attrition bias. From the results in Table S2, we found that the percentage distributions of children across the selected characteristics were similar in the cross-sectional and panel surveys. Only the percentage distribution of children by age and type of community differed by greater than 3% between the two surveys.

### Multivariable analysis

Table 2 represents the multivariable association between the educational wellbeing of children in 2012 and their individual, household and community characteristics during 2005. The result shows that children from the Non-ST group had a significantly higher likelihood of attaining educational wellbeing [coefficient (β): 0.14, 95% CI: (0.07, 0.21)] than their ST counterparts. Child educational wellbeing scores increased among ST and Non-ST children with the growing age. Being stunted at an early age (1–4 years of age) decreased children’s educational wellbeing scores. In 8–11 years, taking private tuition was associated with a significantly higher educational wellbeing score [β: 0.20, CI: (0.15, 0.25)] than children who did not take private tuition. The educational wellbeing score of children increases with the increasing economic gradient of the household. Further, having a female adult with more than 10 years of schooling in a household increases educational wellbeing among both ST [β: 0.28, CI: (−0.03, 0.58)] and Non-ST [β: 0.32, CI: (0.25, 0.39)] children. Children from communities where people solved their problems together [β: 0.05, CI: (0.02, 0.09)] and did not contain domestic violence [β: 0.09, CI: (0.04, 0.14)] had better educational wellbeing than their counterparts. The educational wellbeing score of ST children was higher in Western regions of India than in the Northern region [β: 0.28, CI: (0.02, 0.53)].

**Table 2:** Linear regression models showing the multivariate association between educational wellbeing score and the individual, household and community characteristics of the panel of children aged 1–4 years during IHDS round-I.

Characteristics	Educational wellbeing score of children aged 8–11 years in round-II					
	All children		ST group		Non-ST group	
	Coef	95% CI	Coef	95% CI	Coef	95% CI
**Caste of household head**						
ST group	(Ref)					
Non-ST group	0.14***	(0.07, 0.21)	–	–	–	–
**Age of children (in years)**						
One	(Ref)		(Ref)		(Ref)	
Two	0.21***	(0.16, 0.27)	0.23**	(0.04, 0.42)	0.21***	(0.15, 0.27)
Three	0.37***	(0.32, 0.43)	0.33***	(0.14, 0.52)	0.37***	(0.31, 0.43)
Four	0.49***	(0.43, 0.55)	0.51***	(0.31, 0.72)	0.48***	(0.42, 0.54)
**Gender of the children**						
Female	(Ref)		(Ref)		(Ref)	
Male	0.01	(− 0.03, 0.05)	0.077	(− 0.05, 0.20)	0.00051	(− 0.04, 0.04)
**Stunting status of children**						
Stunted	(Ref)		(Ref)		(Ref)	
Not stunted	0.15***	(0.11, 0.19)	0.14**	(0.01, 0.27)	0.15***	(0.11, 0.19)
**Type of school attended in round-II**						
Government School	(Ref)		(Ref)		(Ref)	
Private School	0.15***	(0.11, 0.20)	0.11	(− 0.11, 0.32)	0.15***	(0.10, 0.20)
**Takes private tuition in round-II**						
No	(Ref)		(Ref)		(Ref)	
Yes	0.20***	(0.15, 0.25)	0.25**	(0.02, 0.49)	0.20***	(0.15, 0.25)
**Place of cooking in household**						
Not in living area	(Ref)		(Ref)		(Ref)	
In living area	− 0.01	(− 0.06, 0.04)	− 0.013	(− 0.15, 0.13)	− 0.0053	(− 0.06, 0.04)
**Type of cooking fuel**						
Solid fuel	(Ref)		(Ref)		(Ref)	
Clean fuel	− 0.01	(− 0.08, 0.07)	− 0.025	(− 0.43, 0.38)	− 0.0041	(− 0.08, 0.07)
**Household sanitation condition**						
Poor	(Ref)		(Ref)		(Ref)	
Average	0.079***	(0.04, 0.12)	0.13	(− 0.03, 0.29)	0.074***	(0.03, 0.12)
Good	0.19***	(0.12, 0.26)	0.27	(− 0.15, 0.70)	0.18***	(0.11, 0.26)
**Water purification in household**						
No	(Ref)		(Ref)		(Ref)	
Yes	0.057**	(0.01, 0.10)	− 0.024	(− 0.18, 0.13)	0.069***	(0.02, 0.12)
**Household wealth quintile**						
Poorest	(Ref)		(Ref)		(Ref)	
Poor	0.13***	(0.07, 0.19)	0.16*	(− 0.02, 0.33)	0.12***	(0.06, 0.19)
Medium	0.28***	(0.21, 0.34)	0.12	(− 0.14, 0.37)	0.28***	(0.21, 0.35)
Rich	0.30***	(0.22, 0.37)	0.20	(− 0.17, 0.58)	0.31***	(0.23, 0.39)
Richest	0.38***	(0.28, 0.47)	0.15	(− 0.31, 0.61)	0.39***	(0.29, 0.49)
**Household poverty status**						
Below poverty line	(Ref)		(Ref)		(Ref)	
Not below poverty line	0.075***	(0.03, 0.12)	0.079	(− 0.09, 0.25)	0.068***	(0.02, 0.11)
**Highest educational level of male adults in household**						
No formal schooling	(Ref)		(Ref)		(Ref)	
Upto 5 years of schooling	0.12***	(0.06, 0.19)	0.020	(− 0.16, 0.20)	0.13***	(0.07, 0.20)
6–10 years of schooling	0.20***	(0.14, 0.26)	0.13	(− 0.06, 0.32)	0.21***	(0.15, 0.27)
More than 10 years of schooling	0.28***	(0.21, 0.34)	0.32***	(0.08, 0.55)	0.28***	(0.21, 0.34)
**Highest educational level of female adults in household**						
No formal schooling	(Ref)		(Ref)		(Ref)	
Upto 5 years of schooling	0.22***	(0.16, 0.28)	0.29***	(0.09, 0.48)	0.21***	(0.15, 0.27)
6–10 years of schooling	0.30***	(0.25, 0.36)	0.32***	(0.10, 0.54)	0.30***	(0.24, 0.36)
More than 10 years of schooling	0.32***	(0.25, 0.39)	0.28*	(− 0.03, 0.58)	0.32***	(0.25, 0.39)
**Gender of household head**						
Male	(Ref)		(Ref)		(Ref)	
Female	0.04	(− 0.04, 0.11)	0.0083	(− 0.27, 0.28)	0.036	(− 0.05, 0.12)
**Religion of household head**						
Hindu	(Ref)		(Ref)		(Ref)	
Muslim	− 0.25***	(− 0.30, − 0.19)	0.0050	(− 0.78, 0.79)	− 0.25***	(− 0.30, − 0.19)
Others	0.05	(− 0.03, 0.13)	− 0.012	(− 0.22, 0.20)	0.059	(− 0.03, 0.15)
**Types of mass media viewed by children**						
None	(Ref)		(Ref)		(Ref)	
One	0.01	(− 0.04, 0.05)	− 0.13*	(− 0.28, 0.02)	0.017	(− 0.03, 0.06)
Two or more	0.02	(− 0.03, 0.07)	− 0.054	(− 0.25, 0.15)	0.025	(− 0.03, 0.08)
**Women’s autonomy in child healthcare in household**						
No	(Ref)		(Ref)		(Ref)	
Yes	0.054**	(0.01, 0.10)	− 0.019	(− 0.18, 0.14)	0.057***	(0.01, 0.10)
**Attack/threat on household**						
Yes	(Ref)		(Ref)		(Ref)	
No	0.22***	(0.11, 0.34)	0.14	(− 0.28, 0.55)	0.24***	(0.12, 0.36)
**Solving community problem**						
Each family individually	(Ref)		(Ref)		(Ref)	
All families together	0.054***	(0.02, 0.09)	0.18***	(0.05, 0.32)	0.040**	(0.00, 0.08)
**Domestic violence in community**						
Yes	(Ref)		(Ref)		(Ref)	
No	0.091***	(0.04, 0.14)	0.044	(− 0.13, 0.22)	0.10***	(0.05, 0.16)
**Type of community**						
Urban	(Ref)		(Ref)		(Ref)	
Rural	0.02	(− 0.04, 0.07)	− 0.099	(− 0.37, 0.17)	0.028	(− 0.03, 0.08)
**Country regions**						
Northern	(Ref)		(Ref)		(Ref)	
North Eastern	− 0.13*	(− 0.26, 0.01)	0.11	(− 0.26, 0.48)	− 0.19**	(− 0.35, − 0.03)
Central	0.036	(− 0.03, 0.10)	− 0.18	(− 0.41, 0.05)	0.084**	(0.01, 0.15)
Eastern	0.024	(− 0.04, 0.09)	− 0.12	(− 0.38, 0.14)	0.041	(− 0.02, 0.10)
Western	0.0044	(− 0.06, 0.07)	0.28**	(0.02, 0.53)	− 0.035	(− 0.10, 0.03)
Southern	− 0.057*	(− 0.12, 0.00)	0.14	(− 0.14, 0.42)	− 0.073**	(− 0.13, − 0.01)
**Adjusted R-squared**	0.248		0.231		0.238	
**Analytical sample size**	8,611		761		7,850	

(a) *Coef* coefficient, *CI* confidence interval, (Ref) reference category; (b) statistical significance denoted by asterisks where *p-value < 0.1, **p-value < 0.05 and ***p-value < 0.01.

### Decomposing the caste inequality in educational wellbeing score

Table 3 reveals the overall Blinder-Oaxaca decomposition of educational wellbeing scores among India’s ST and Non-ST children. While adjusting endowment levels of Non-ST children with that of their ST counterparts would increase the ST children’s educational wellbeing score by 79.1%, 20.9% of differences were left unexplained. Further, we presented a detailed decomposition analysis of the inequality in educational wellbeing scores among India’s ST and Non-ST children (Table 4). Among 79.1% explained share, household-level variables of wealth quintile (24.3%), highest educational level of adult males (13.7%) and females (13.1%) in the families has a prominent contribution to ST and Non-ST children’s educational wellbeing inequality. Among the child-related characteristics, taking private tuitions (6.9%), school attending status (6.2%), and stunting status (2.5%) had significantly higher contributions to caste inequality. Additionally, household sanitation conditions and poverty status contribute to children’s educational wellbeing inequality. Domestic violence in the community (−0.8%) shows a significantly negative influence on the inequality of educational wellbeing scores. This negative value indicates that ST children experience higher educational wellbeing scores in a community with no domestic violence, and if we eliminate this advantage, it would further deteriorate their children’s educational wellbeing scores.

**Table 3:** Overall Blinder-Oaxaca decomposition of the caste differential in educational wellbeing score of the panel of children during IHDS round-II.

Component	Caste differential in educational wellbeing		
	Coefficient	95% CI	Percent
Explained difference (E)	−0.360***	(−0.398, −0.321)	79.1
Unexplained difference (U)	−0.095***	(−0.163, −0.027)	20.9
Total difference (T)	−0.454***	(−0.528, −0.381)	

(a) *CI* confidence interval; (b) statistical significance denoted by asterisks where *p-value < 0.1, **p-value < 0.05 and ***p-value < 0.01.

**Table 4:** Detailed Blinder-Oaxaca decomposition of the caste differential in educational wellbeing score of the panel of children during IHDS round-II.

Characteristics	Caste differential in educational wellbeing among children in round-II					
	Explained component (E)			Unexplained component (U)		
	Coefficient	95% CI	Percent	Coefficient	95% CI	Percent
Age of children (in years)	−0.003	(−0.014, 0.009)	0.6	0.018	(−0.142, 0.178)	−3.9
Gender of the children	0.000	(0.000, 0.000)	0.0	0.042	(−0.027, 0.111)	−9.3
Stunting status of children	−0.012***	(−0.018, −0.005)	2.5	−0.009	(−0.072, 0.054)	2.0
Type of school attended in round-II	−0.028***	(−0.039, −0.017)	6.2	−0.042	(−0.273, 0.189)	9.3
Takes private tuition in round-II	−0.031***	(−0.039, −0.023)	6.9	−0.006	(−0.030, 0.017)	1.4
Place of cooking in household	0.001	(−0.007, 0.010)	−0.3	−0.016	(−0.068, 0.035)	3.5
Type of cooking fuel	0.002	(−0.004, 0.008)	−0.4	−0.001	(−0.016, 0.015)	0.1
Household sanitation condition	−0.026***	(−0.036, −0.016)	5.7	0.003	(−0.044, 0.050)	−0.7
Water purification in household	0.000	(−0.001, 0.002)	−0.1	−0.035	(−0.081, 0.012)	7.6
Household wealth quintile	−0.111***	(−0.137, −0.084)	24.3	−0.023	(−0.200, 0.154)	5.1
Household poverty status	−0.022***	(−0.036, −0.008)	4.9	0.035	(−0.033, 0.103)	−7.6
Highest educational level of male adults in household	−0.062***	(−0.078, −0.047)	13.7	−0.009	(−0.102, 0.083)	2.1
Highest educational level of female adults in household	−0.060***	(−0.073, −0.046)	13.1	0.029	(−0.030, 0.088)	−6.4
Gender of household head	0.000	(−0.001, 0.001)	0.0	0.039	(−0.232, 0.310)	−8.6
Religion of household head	−0.010***	(−0.017, −0.004)	2.3	0.126**	(0.003, 0.249)	−27.7
Types of mass media viewed by children	−0.004	(−0.011, 0.003)	0.8	−0.038	(−0.095, 0.019)	8.4
Women's autonomy in child healthcare in household	−0.003**	(−0.007, 0.000)	0.8	0.003	(−0.034, 0.040)	−0.7
Attack/threat on household	0.000	(−0.002, 0.003)	−0.1	−0.055	(−0.410, 0.300)	12.1
Solving community problem	−0.001	(−0.003, 0.001)	0.2	0.118***	(0.039, 0.197)	−26.0
Domestic violence in community	0.003**	(0.000, 0.007)	−0.8	−0.017	(−0.048, 0.015)	3.7
Type of community	0.008	(−0.002, 0.017)	−1.7	−0.108	(−0.339, 0.123)	23.8
Country regions	−0.002	(−0.006, 0.003)	0.3	0.185**	(0.030, 0.339)	−40.6
Constant	–	–	–	−0.334	(−0.979, 0.312)	73.4

(a) *CI* confidence interval; (b) statistical significance denoted by asterisks where *p-value < 0.1, **p-value < 0.05 and ***p-value < 0.01.

### Sensitivity analysis of decomposition estimates to the categorization of comparison groups

Table 5 shows the decomposition of the educational wellbeing gap among children in ST and Non-ST, ST and SC, ST and OBC, and ST and Others groups, respectively. We find that the direction of contribution is the same across all the statically significant contributors in the four decomposition estimates. The explained educational wellbeing difference between ST and Non-ST groups is similar for the ST and OBC, and ST and Others groups. The magnitude of the percentage contribution of each statistically significant contributor varies across the four decomposition estimates. However, the difference in the percentage contribution is not more than 5% in the contributors across the four decomposition estimates.

**Table 5:** Blinder-Oaxaca decomposition of the caste differential in educational wellbeing score between ST and Non-ST, ST and SC, ST and OBC, and ST and Others children for sensitivity analysis.

Characteristics	Caste differential in educational wellbeing among children in round-II							
	Explained difference of ST and Non-ST group		Explained difference of ST and SC group		Explained difference of ST and OBC group		Explained difference of ST and Others group	
	Coefficient	Percent	Coefficient	Percent	Coefficient	Percent	Coefficient	Percent
Age of children (in years)	−0.003	0.6	0.005	−1.7	−0.005	1.3	−0.005	0.7
Gender of the children	0.000	0.0	0.001	−0.5	0.000	0.0	0.000	0.0
Stunting satus of children	−0.012*	2.5	−0.006	2.0	−0.010*	2.4	−0.018*	2.7
Type of school attended in round-II	−0.028*	6.2	−0.011*	3.5	−0.039*	9.8	−0.025	3.7
Takes private tuition in round-II	−0.031*	6.9	−0.029*	9.2	−0.017*	4.3	−0.054*	8.2
Place of cooking in household	0.001	−0.3	−0.008	2.6	0.004	−1.1	0.005	−0.7
Type of cooking fuel	0.002	−0.4	0.001	−0.2	0.007*	−1.8	−0.009	1.4
Household sanitation condition	−0.026*	5.7	−0.011*	3.4	−0.022*	5.6	−0.049*	7.3
Water purification in household	0.000	−0.1	0.004	−1.2	0.000	−0.1	0.002	−0.3
Household wealth quintile	−0.111*	24.3	−0.109*	34.9	−0.092*	22.8	−0.100*	15.0
Household poverty status	−0.022*	4.9	−0.007	2.1	−0.022*	5.5	−0.069*	10.3
Highest educational level of male adults in household	−0.062*	13.7	−0.025*	7.9	−0.057*	14.2	−0.121*	18.1
Highest educational level of female adults in household	−0.060*	13.1	−0.018*	5.6	−0.057*	14.3	−0.092*	13.9
Gender of household head	0.000	0.0	0.000	0.0	0.000	0.0	0.000	0.0
Religion of household head	−0.010*	2.3	0.013	−4.1	−0.020*	5.0	−0.001	0.2
Types of mass media viewed by children	−0.004	0.8	0.002	−0.5	−0.005	1.4	0.002	−0.3
Women's autonomy in child healthcare in household	−0.003*	0.8	−0.003	0.9	−0.002	0.4	−0.009*	1.3
Attack/threat on household	0.000	−0.1	0.000	−0.1	0.001	−0.3	−0.001	0.1
Solving community problem	−0.001	0.2	−0.006	1.8	0.001	−0.1	−0.002	0.4
Domestic violence in community	0.003*	−0.8	0.003	−1.1	0.002	−0.5	0.000	−0.1
Type of community	0.008	−1.7	0.011	−3.5	0.002	−0.6	−0.002	0.3
Country regions	−0.002	0.3	0.009	−3.0	0.001	−0.2	−0.004	0.6
Explained difference (E)	−0.360*	79.1	−0.181*	57.8	−0.330*	82.3	−0.552*	82.9
Unexplained difference (U)	−0.095*	20.9	−0.132*	42.2	−0.071	17.7	−0.114*	17.1
Total difference (T)	−0.454*		−0.314*		−0.401*		−0.666*	

(a) Statistical significance denoted by asterisks where *p-value < 0.05.

### Ethics declarations

The present study utilized a publicly available secondary dataset with no information that would lead to the identification of the respondents. IHDS obtained the consent of respondents before data collection. Therefore, no ethical approval was necessary. All survey methods were performed following the relevant guidelines and regulations.

## Discussion

Understanding the power of education in changing the lives of individuals, families and communities, the government of India has made a constant effort to bring children to schools and provide primary education. However, before celebrating the success of bridging the schooling gap in tribal (ST) and non-tribal (Non-ST) children, it is essential to determine the quality of education these children received in the past few years. Sadly, the present study shows a challenging face of the education system, where the educational wellbeing score of tribal children is significantly lower than their non-tribal counterparts. The salient findings of the study and their explanations are as follows:

First, although the government has tried to eradicate caste-based discrimination in the education system, it is still prevalent with the tribal population at the receiving end. This finding is supported by the multivariable regression and decomposition analysis results. Such a situation may arise due to the unavailability of good schools in the community and qualified teachers. Past evidence has shown that their reading, writing, and mathematical competence was shallow even if the tribal children were attending schools. Curriculum and communication play an essential role in preventing children’s educational wellbeing. It has been observed that the inclusion of local culture, folklore and history, and the local dialect in the curriculum builds confidence in tribal children. Further, interpreting through paintings, music, and storytelling can improve their educational wellbeing as they are common in their culture.

Second, early life stunting status can hinder the educational wellbeing of children. Consistent with the present study, an Indian study showed that child nutritional status affects their physical, cognitive and language development^16^. Moreover, the present study confirms that the child’s educational wellbeing depends on their type of school and private tuition. Besides, these child characteristics—stunting status in early life, private schooling, and private tuitions largely contribute to the educational wellbeing inequality between tribal (ST) and non-tribal (Non-ST) children. Third, early life circumstances like household wealth index and poverty status are significant hindrances to children’s educational wellbeing, as the spending on education can be done only in those households which can pay^39^. Most tribal households cannot fulfill their basic living needs, so education becomes their secondary priority. Even if the government had introduced free education and mid-day meal schemes for bringing the tribal children to schools, the financial constraints of households would restrict them from completing their education. Studies have shown that absenteeism and dropout are higher among tribal children, especially during crop cultivation. This situation can leave the children behind in the classroom compared to other regularly attending schools. Fourth, parents’ education or the education of household members can also affect the educational wellbeing of children. Since uneducated elders in the household cannot help the children efficiently, there is past evidence that parent involvement has a commendable role in a child’s educational achievement^40^.

To the best of our knowledge, the current study is among the few studies examining inequality in India’s educational wellbeing scores of tribal and non-tribal children. Further, using the decomposition analysis, the study shows the contribution of early life circumstances to such inequality. We know that the early childhood period represents the development pedestal for the later years. Children’s exposure to physiological and socioeconomic stress during this period gets manifested as reduced educational wellbeing in the long run^16,20,41^. Therefore, cross-sectional studies examining the relationship between educational wellbeing determinants will misestimate the effect. The panel nature of this study helps us point out the role of individual, household and community factors of children aged 1–4 years (early childhood period) behind the differential in educational wellbeing in tribal and non-tribal children when they become 8–11 years.

Moreover, the study’s findings did not suffer from attrition bias as the demographic and socioeconomic distribution of children in the cross-sectional and panel surveys during the baseline period were similar. This finding is similar to other studies that have used the IHDS panel dataset^16,25,40^. Additionally, the decomposition estimates were not sensitive to the heterogeneity in the non-tribal group. The sensitivity analysis revealed that the decomposition estimates were robust to the categorization of comparison groups. However, the study has its shortcomings. This study did not provide any causal inference. Further, due to the requirement of including a nationally representative panel dataset to show the contribution of early life circumstances, we have to use data from 2005 and 2012. Therefore, readers need to be cautious of the survey date while interpreting this study’s findings.

## Conclusion

The missing focus on the minority groups excluded these communities from education participation. Historically, tribal children faced rejection and discrimination in terms of their backwardness. Such discrimination can be seen in inequality in their educational wellbeing due to their early life circumstances. Commendable progress has brought tribal children to schools in the past few years. Still, efforts should also be made towards reducing their discontinuation and improving their quality of education which can improve their educational wellbeing. Quality education refers to both qualities of the school infrastructure, teacher and the learning process. Inclusion of an interactive curriculum based on their culture with proper communication at basic levels can help improve children’s educational wellbeing. Besides these factors, policies should also focus on providing targeted interventions during the early childhood period of tribal children by improving their household conditions, sensitization of parents and the community about educational opportunities and advantages during their initial years, and creating a peaceful and healthy community. Notably, early childhood conditions can be improved by providing targeted benefits to ST children through existing nutrition-security and wellbeing programs (Integrated Child Development Services, Antyodaya Anna Yojana and Poshan Abhiyan) of the Indian government.

## Supplementary Information

Supplementary Tables.

## Data Availability

The study utilizes a secondary source of data that is freely available in the public domain from the Inter-University Consortium for Political and Social Research (ICPSR) data repository (https://www.icpsr.umich.edu/web/DSDR/series/507).
