# Supplementary material for: Early childhood circumstances and educational wellbeing inequality among tribal and non-tribal children in India: evidence from a panel study
Source: Sci Rep. 2022 Jun 14;12:9839. doi: 10.1038/s41598-022-13889-5 (PMC9197972; doi:10.1038/s41598-022-13889-5)
Supplement: Supplementary file 1 — Supplementary Tables. [file 41598_2022_13889_MOESM1_ESM.docx]

**Supplementary Tables**

**Table S1: Results obtained from the Principal Component Factor Analysis (PCFA) of the indicators of educational wellbeing**

| **Scale** |  | **Items** | **Factor Loading** | **KMO Test** |
| --- | --- | --- | --- | --- |
| **Educational wellbeing (1st Factor)** |  |  |  |  |
| Eigen value = 2.20 |  | Reading skill | 0.87 | 0.70 |
| Explained variance = 73.4% |  | Mathematical skill | 0.86 | 0.71 |
| Cronbach's alpha = 0.82 |  | Writing skill | 0.84 | 0.74 |

**Table S2: Results obtained from the Principal Component Factor Analysis (PCFA) of the indicators of cognitive wellbeing**

| **Characteristics** | **Children aged 1-4 years during IHDS baseline survey** | | | | | | |
| --- | --- | --- | --- | --- | --- | --- | --- |
|  | **Cross-sectional Dataset** | |  | **Panel Dataset** | |  | **Absolute Difference (%)** |
|  | **N** | **%** |  | **N** | **%** |  |  |
| **Caste of the household head** |  |  |  |  |  |  |  |
| Scheduled Tribes | 1,486 | 9.1 |  | 737 | 8.6 |  | 0.5 |
| Scheduled Castes | 3,578 | 21.8 |  | 1,955 | 22.7 |  | 0.9 |
| Other Backward Classes | 6,799 | 41.4 |  | 3,557 | 41.3 |  | 0.1 |
| Others | 4,541 | 27.7 |  | 2,362 | 27.4 |  | 0.3 |
| **Age of the child (in years)** |  |  |  |  |  |  |  |
| One | 3,502 | 21.3 |  | 1,496 | 17.4 |  | 3.9 |
| Two | 4,215 | 25.7 |  | 2,474 | 28.7 |  | 3.0 |
| Three | 4,499 | 27.4 |  | 2,671 | 31.0 |  | 3.6 |
| Four | 4,188 | 25.5 |  | 1,970 | 22.9 |  | 2.6 |
| **Gender of the children** |  |  |  |  |  |  |  |
| Male | 8,531 | 52.0 |  | 4,529 | 52.6 |  | 0.6 |
| Female | 7,873 | 48.0 |  | 4,082 | 47.4 |  | 0.6 |
| **Type of community** |  |  |  |  |  |  |  |
| Rural | 11,492 | 70.1 |  | 6,330 | 73.5 |  | 3.4 |
| Urban | 4,912 | 29.9 |  | 2,281 | 26.5 |  | 3.4 |
| **Religion of the household head** |  |  |  |  |  |  |  |
| Hindu | 12,860 | 78.4 |  | 6,791 | 78.9 |  | 0.5 |
| Muslim | 2,490 | 15.2 |  | 1,293 | 15.0 |  | 0.2 |
| Others | 1,054 | 6.4 |  | 527 | 6.1 |  | 0.3 |
| **Household below poverty line** |  |  |  |  |  |  |  |
| Yes | 5,258 | 32.1 |  | 2,782 | 32.3 |  | 0.2 |
| No | 11,146 | 67.9 |  | 5,829 | 67.7 |  | 0.2 |
| **Wealth quintile** |  |  |  |  |  |  |  |
| Poorest | 3,424 | 20.9 |  | 1,884 | 21.9 |  | 1.0 |
| Poor | 3,161 | 19.3 |  | 1,694 | 19.7 |  | 0.4 |
| Medium | 3,251 | 19.8 |  | 1,719 | 20.0 |  | 0.2 |
| Rich | 3,393 | 20.7 |  | 1,759 | 20.4 |  | 0.3 |
| Richest | 3,175 | 19.4 |  | 1,555 | 18.1 |  | 1.3 |
| **Highest educational level of  male adult (21+ years)** |  |  |  |  |  |  |  |
| No formal schooling | 3,485 | 21.7 |  | 1,989 | 23.1 |  | 1.4 |
| Upto 5 years of schooling | 2,508 | 15.6 |  | 1,343 | 15.6 |  | 0.0 |
| 6-10 years of schooling | 4,276 | 26.6 |  | 2,307 | 26.8 |  | 0.2 |
| More than 10 years of schooling | 5,791 | 36.1 |  | 2,972 | 34.5 |  | 1.6 |
| **Highest educational level of  female adult (21+ years)** |  |  |  |  |  |  |  |
| No formal schooling | 7,260 | 44.5 |  | 3,893 | 45.2 |  | 0.7 |
| Upto 5 years of schooling | 2,325 | 14.2 |  | 1,273 | 14.8 |  | 0.6 |
| 6-10 years of schooling | 3,264 | 20.0 |  | 1,727 | 20.1 |  | 0.1 |
| More than 10 years of schooling | 3,467 | 21.2 |  | 1,718 | 20.0 |  | 1.2 |
| **Overall** | **16,404** | **100** |  | **8,611** | **100** |  | **0** |

**Note – (a) N: Sample count, %: Column percentage.**
